# Modified Black Soldier Fly Larva Fat in Broiler Diet: Effects on Performance, Carcass Traits, Blood Parameters, Histomorphological Features and Gut Microbiota

**DOI:** 10.3390/ani11061837

**Published:** 2021-06-21

**Authors:** Sihem Dabbou, Angelo Lauwaerts, Ilario Ferrocino, Ilaria Biasato, Federico Sirri, Marco Zampiga, Stefania Bergagna, Giulia Pagliasso, Marta Gariglio, Elena Colombino, Carlos Garcés Narro, Francesco Gai, Maria Teresa Capucchio, Laura Gasco, Luca Cocolin, Achille Schiavone

**Affiliations:** 1 Center Agriculture Food Environment (C3A), University of Trento, Via E. Mach 1, 38010 San Michele all’Adige, Italy; sihem.dabbou@unitn.it; 2 Salka Valka BV, Slachthuisstraat 59, 9000 Gent, Belgium; angelo@salkavalka.eu; 3 Department of Agricultural, Forest and Food Sciences, University of Turin, Largo Paolo Braccini 2, Grugliasco, 10095 Turin, Italy; ilaria.biasato@unito.it (I.B.); laura.gasco@unito.it (L.G.); lucasimone.cocolin@unito.it (L.C.); 4 Department of Agricultural and Food Sciences, Alma Mater Studiorum—University of Bologna, Via del Florio 2, Ozzano dell’Emilia, 40064 Bologna, Italy; federico.sirri@unibo.it (F.S.); marco.zampiga2@unibo.it (M.Z.); 5 Veterinary Medical Research Institute for Piemonte, Liguria and Valle d’Aosta, Via Bologna 148, 10154 Turin, Italy; stefania.Bergagna@izsto.it (S.B.); giulia.pagliasso@izsto.it (G.P.); 6 Department of Veterinary Sciences, University of Turin, Largo Paolo Braccini 2, Grugliasco, 10095 Turin, Italy; marta.gariglio@unito.it (M.G.); elena.colombino@unito.it (E.C.); mariateresa.capucchio@unito.it (M.T.C.); achille.schiavone@unito.it (A.S.); 7 Faculty of Veterinary Medicine, Universidad CEU Cardenal Herrera, CEU Universities, Alfara de Patriarca, E-46115 Valencia, Spain; cgarces@uchceu.es; 8 Institute of Science of Food Production, National Research Council, Largo Paolo Braccini 2, Grugliasco, 10095 Turin, Italy; francesco.gai@ispa.cnr.it

**Keywords:** modified black soldier fly larvae fat, chickens, microbiota, meat quality

## Abstract

**Simple Summary:**

Black soldier fly (*Hermetia illucens* L.; BSF) is gaining interest as a functional feed additive, due to the high amount of medium-chain fatty acids (MCFAs) and monoglycerides, which provide antimicrobial activities and stimulate gastrointestinal health through inhibition of potentially pathogenic bacteria. The present study evaluated the effect of BSF and modified BSF larvae fat in broiler chicken’s diet. Overall results were comparable among the studied diets, suggesting that modified BSF larvae fat showed a positive modulation of fecal microbiota by a positive reduction in potentially pathogenic bacteria such as *Clostridium* and *Corynebacterium,* without affecting intestinal morphology or showing any adverse histopathological alternations.

**Abstract:**

In this study, a total of 200 male broiler chickens (Ross 308) were assigned to four dietary treatments (5 pens/treatment and 10 birds/pen) for two feeding phases: starter (0–11 days of age) and grower-finisher (11–33 days of age). A basal diet containing soy oil (SO) as added fat was used as control group (C), tested against three experimental diets where the SO was partially substituted by BSF larvae fat (BSF) or one of two types of modified BSF larvae fat (MBSF1 and MBSF2, respectively). The two modified BSF larvae fats had a high and low ratio of monobutyrin to monoglycerides of medium chain fatty acid, respectively. Diet did not influence the growth or slaughter performance, pH, color, or the chemical composition of breast and thigh muscles, gut morphometric indices, or histopathological alterations in all the organs. As far as fecal microbiota are concerned, MBSF1 and MBSF2 diets reduced the presence of *Clostridium* and *Corynebacterium*, which can frequently cause infection in poultry. In conclusion, modified BSF larva fat may positively modulate the fecal microbiota of broiler chickens without influencing the growth performance and intestinal morphology or showing any adverse histopathological alternations.

## 1. Introduction

Nowadays, the poultry industry is one the most important and fast-growing among livestock sectors. However, ensuring a sufficient quantity of poultry meat to satisfy the global population increase is one of the greatest challenges. The use of antibiotics under long-term administration has been a strategy to prevent health problems and support productive performance. However, antibiotics can modify intestinal microbiota and increase antibiotic-resistant pathogens in poultry [1]; furthermore, they are currently banned in the EU due to the resistance issue. In this context, several alternatives strategies such as feed additives, prebiotics, probiotics, and organic acids were used to modulate the intestinal microbiota, to develop a healthy digestive system in animals without extensive use of antibiotics, and consequently, to promote the growth performance of poultry [2]. Fats and oils are the main sources of energy as they have the highest caloric value among all ingredients. For this reason, the use of supplemental fats in feed formulation is a widespread practice for meeting both the energy and also essential fatty acid (FA) requirements.

Fat derived from black soldier fly larvae meal (*Hermetia illucens* L. BSF) was recently studied and used in the formulation of livestock and aquaculture diets as a substitution for the commonly used lipid sources, such as rendered fat and vegetable oils, in order to improve animal performance and meat quality and to maintain ecological sustainability [3,4,5,6,7,8,9,10]. BSF larvae fat is rich in medium chain fatty acids (MCFA), and lauric acid (C12:0) is a major constituent (up to 52%) [11]. The FA composition of BSF fat is similar to that of palm kernel oil and coconut fat, and therefore it can be used in animal feed. The BSF FA composition is influenced by the larval substrate [11,12,13], weight, and stage of development [11,14,15]. Ewald et al. [11] showed a positive correlation between larval weight and the percentage of lauric acid and total saturated FAs (SFA) in the larvae and suggested that SFA accumulate most as the larvae gain weight.

In livestock, dietary MCFA, and in particular lauric acid, have a positive effect on gut microbiota [16,17]; therefore, it was suggested to focus on the augmentation of lauric acid in insect larvae [11]. MCFA can be used as an energy source, improving energy availability without increasing the deposition of lipids [3]. Moreover, in livestock, the MCFA have an antimicrobial effect thus can stimulate gastrointestinal tract health through inhibition of potentially pathogenic bacteria [16,17]. Van Immerseel et al. [18] reported that, in poultry, MCFA have the greatest antimicrobial activity against *Salmonella*, which causes gastroenteritis. MCFA also have an inhibitory effect mediated by *Lactobacillus* [19]. Moreover, it was highlighted that the supplementation of 3% MCFA as a bactericidal agent reduced the number of bacteria in broiler chickens [20]. van Gerwe et al. [21] stated that the addition of a MCFA mixture to the feed at 1% reduces the colonization of broiler with *Campylobacter jejuni*. On the other hand, MCFA can improve nutrient absorption and intestinal morphology of broilers with an increase of villi length and the villi:crypt ratio in the duodenum and jejunum [22]. Combinations of the monoglyceride of butyric acid (MB) are considered as an alternative to GPAs in the diet of broiler chickens [23]. Butyric acid and MCFA, especially lauric acid, have been demonstrated to reduce necrotic enteritis induced by *Clostridium perfringens* [24]. Zeiger et al. [25] suggests that lauric acid as a feed additive has the potential to improve food safety by reducing the numbers of *Campylobacter coli* in broiler meat. Fortuoso et al. [26] showed that glycerol monolaurate, known as lauric acid, in the diets of broiler chickens has a potent antimicrobial effect, growth promoter capacity, and lack of toxicity. From all forms of MCFA, monoglycerides show the highest antimicrobial activity in vitro [27]. The lipolysis of different monoglycerides has been studied in detail in vitro by Martin et al. [28] and Sek et al. [29]. In particular, 1-monoglycerides are readily hydrolyzed. Moreover, a synergy between MCFA and the corresponding monoglycerides has been demonstrated [30]. When monoglycerides pass through the gastrointestinal tract, the lipase excreted at different points will result in a common presence in vivo of monoglycerides and the corresponding acids [31]. It is probable that the observed synergies in vitro will therefore also play an active role in the gastrointestinal tract in vivo. Monoglycerides may hence be a preferred embodiment for the delivery of MCFA. Schiavone et al. [5] recently demonstrated that the use of BSF larva fat in the diets for finisher broiler chickens has no adverse effects on growth performance, blood profile, or histological features, also allowing the preservation of physiological gut morphological development. In a recent study, Sypniewski et al. [9] showed that the addition of BSF fat as a substitute for soybean oil in turkey diets, significantly reduced the numbers of potentially pathogenic bacteria and decreased the level of selected immune traits, i.e., IL-6 and TNF-α, which are related to GIT inflammation, without any adverse effect on growth performance, nutrient digestibility, GIT morphology, or quality of the breast and thigh muscles.

Based on the above reported background, the aim of this study was to investigate how the standard BSF larva fat and modified BSF larva fats, used as feed additive, have an effect on growth performance, meat quality, blood parameters, intestinal morphology, histological features, and gut microbiota of broiler chickens.

## 2. Materials and Methods

### 2.1. Birds and Diets

The study was carried out at the poultry facility of the University of Turin (Italy). The poultry house was 7 m wide × 50 m long × 7 m high, and was equipped with a waterproof floor and walls, completely covered by tiles, and had an automatic ventilation system. A total of 200, 1-day-old, male broiler chickens (ROSS 308) were housed in 20 pens and randomly allotted to 4 dietary treatments, each group consisting of 5 pens as replicates with 10 birds per pen (average live weight (LW): 45.29 ± 3.03 g). The birds were individually marked by a wing tag. Each pen was 1.20 m wide × 2.20 m long and was covered with rice hulls as litter. During the first 3 weeks, the animals were warmed by infrared lamps. The lighting schedule was 23 h light and 1 h darkness until day 3 of the trial, and thereafter the dark period was gradually increased to 6 h and maintained constantly until slaughtering. The environmental parameters were monitored daily during the whole period of the trial. All chicks were vaccinated against infectious bronchitis, Gumboro disease, and Newcastle disease at the hatchery.

Four experimental diets were formulated for two feeding phases: from 1 to 11 d (starter period), and from 11 d to 33 d (grower-finisher period). A basal diet containing soybean oil (SO) as added fat served as control group (C) and was tested against 3 experimental diets where SO was partially substituted by BSF larvae fat (BSF diet) or 1 of 2 types of modified BSF larvae fat (MBSF1 and MBSF2 diets, respectively). The BSF larvae fat is the fat extracted from BSF larvae, as commercially available from PROTIX (Dongen, The Netherlands). This BSF fat was treated with an excess of glycerol to catalytically convert the triglycerides into monoglycerides. Additionally, glycerol monobutyrin was added to this modified fat in 2 different concentrations:

(i) MBSF1 was supplemented with 8–9% glycerol monobutyrin to obtain an equimolar content of monobutyrin and monolaurin;
(ii) MBSF2 was supplemented with 21–22% glycerol monobutyrin such that the amount of FA was equimolar to the amount of added monobutyrin in the resulting product.

Both MBSF1 and MBSF2 are prototypes of a product to maintain optimal gut health in broilers.

### 2.2. Chemical Composition and Fatty Acid Profile of the Experimental Diets

The samples of feed were ground through a 1-mm screen using a cutting mill (MLI 204; Bühler AG, Uzwil, Switzerland). The dry matter (DM) content was determined by drying the samples at 103 °C to constant weight. Ash content was determined by muffle furnace incineration (942.05), crude protein (CP; N × 6.25) was measured with Kjeldahl method (2001.11), and ether extract (EE) quantified by ether extraction (920.39) according to the AOAC [32] standard procedures. Ingredients and chemical composition of the experimental diets are shown in Table 1. FAs were determined as previously reported by Glass and Cristopherson [33] using a Shimadzu GC17A gas chromatograph (Shimadzu Corporation, Tokyo, Japan) with a WP-4 Shimadzu integration system, equipped with a Varian CPSIL88 capillary column (100 m long, 0.25 mm i.d., 0.20 mm thick film) (Varian, Walnut Creek, CA, USA) and a flame ionization detector, and expressed as a percentage of each individual FAME per total FAME detected (Table 2).

### 2.3. Growth Performance

The LWs of birds were recorded individually at their arrival and at the end of each feeding phase. Average daily feed intake (ADFI), average daily weight gain (ADG), and feed conversion ratio (FCR) were calculated for each experimental group per feeding phase and for the entire rearing period. All measurements were performed at a pen level. All weightings were performed using electronic scales with an accuracy of 0.1 g (Signum, Sartorius, Bovenden, Germany).

### 2.4. Blood Parameters

Blood samples were collected, at slaughtering, from the jugular vein of 15 birds (3 animals per pen) per feeding group and were placed into serum-separating tubes. The tubes were left in a standing position, at room temperature, for approximately 2 h, until the formation of a blood clot. Subsequently, the tubes were centrifuged at 2500g for 10 min at 4 °C and the obtained serum was immediately frozen at −80 °C pending analysis. The concentrations of albumin (ALB), uric acid, creatinine, alanine amino transferase (ALT), aspartate aminotransferase (AST), alkaline phosphatase (ALP), gamma-glutamyl transpeptidase (GGT), total cholesterol, high-density lipoprotein (HDL), low-density lipoprotein (LDL), triglycerides, total proteins, calcium, iron, magnesium, phosphorus, sodium, chlorine, and potassium were measured using an automated system photometer (I-Lab Aries Chemical Analyzer—Instrumentation Laboratory) [34].

### 2.5. Slaughter Procedures and Muscle Sampling

Sixty animals (15 per diet, 3 birds/pen selected to be representative of the average final LW in each pen) were individually identified with a shank ring and weighed at 33 days of age. After a feed withdrawal of 12 h, the animals were slaughtered in a commercial abattoir according to the standard EU regulations (1099/2009). Plucked and eviscerated carcasses were obtained after removing the head, neck and feet. The spleen, liver, bursa of Fabricius, heart, intestine, gizzard (muscular stomach), and proventriculus (glandular stomach) weights were immediately recorded and expressed as a percentage of the slaughter weight (SW; recorded immediately before slaughtering). The carcasses were stored at +4 °C for 24 h. The chilled carcass (CC) weight was registered, and the CC yields were calculated as a percentage of SW. The breast and thighs were then excised, and the weights were expressed as percentages of the CC weight.

The pH_24_ was assessed in duplicate on the *Pectoralis major* muscle on the right side of the breast and on the *Biceps femoris* muscle on the right thigh. In particular, the pH of the *Pectoralis major* and *Biceps femoris* muscles was evaluated by means of a pH meter (Crison, Crison Instruments, SA, Alella, Spain) equipped with a specific electrode suitable for meat penetration. The meat color in terms of lightness (L*), redness (a*) and yellowness (b*) indices [35] were measured only on the *Pectoralis major* muscle using a portable Chroma Meter CR-400 Konica Minolta Sensing colorimeter (Minolta Sensing Inc., Osaka, Japan).

### 2.6. Chemical Composition of Meat

Breast and thigh meat chemical composition was evaluated on 15 animals per treatment. The left thigh was skinned and entirely deboned to separate the bones and cartilage from the edible meat. Additionally, the left breast was taken. Both thigh and breast samples were individually vacuum packed and stored at −20 °C until chemical analysis. The moisture and ash content were determined according to the Association of Official Analytical Chemists [36] procedure. Proteins were determined using the standard Kjeldahl copper catalyst method [33]. Total lipids were measured using modification of the chloroform:methanol procedure described by Folch et al. [37].

### 2.7. Histomorphological Investigations

At the end of the trial, 15 birds (3 per pen) per feeding group were submitted to anatomo-pathological investigations. Intestinal segment samples (approximately 5 cm in length) of duodenum, jejunum, ileum, and caecum were excised and flushed with 0.9% saline solution to remove all the content. The collected segments of intestine were the loop of the duodenum, the tract before Meckel’s diverticulum (jejunum), the tract before the ileocolic junction (ileum), and the apex of caecum. Samples of glandular stomach (proventriculus), liver, spleen, thymus, and bursa of Fabricius were also collected. Gut segments and organ samples were fixed in 10% buffered formalin solution for at least 7 days for morphometric analysis (gut segments) and histopathological examination (gut and organ samples). Tissues were routinely embedded in paraffin wax blocks, sectioned at 5 μm thickness, mounted on glass slides, and stained with hematoxylin and eosin (HE). The evaluated morphometric indices were villus height (Vh, from the tip of the villus to the crypt), crypt depth (Cd, from the base of the villus to the submucosa), and the villus height to crypt depth (Vh/Cd) ratio [38]. Morphometric analyses were performed on 10 well-oriented and intact villi and 10 crypts chosen from duodenum, jejunum, and ileum [39]. The observed histopathological findings were evaluated using a semi-quantitative scoring system as follows: absent (score = 0), mild (score = 1), moderate (score = 2) and severe (score = 3). Gut histopathological findings were separately assessed for mucosa (inflammatory infiltrates) and submucosa (inflammatory infiltrates and gut-associated lymphoid tissue (GALT) activation) for each segment. The total score of each gut segment was obtained by adding up the mucosa and submucosa scores, while the total score of each bird was represented by the mean value of the duodenum, jejunum, ileum, and caecum scores.

### 2.8. DNA Extraction and 16S Metataxonomic Approach

In order to observe the development and dynamics of bacterial communities, at 0, 11, and 33 days of age, all birds of each pen were removed from pens and housed in wire-mesh cages (100 × 50 cm width × length) for 30 min per day to collect fresh excreta samples, according to the procedure described by Dabbou et al. [40]. In particular, 5 pooled fecal samples were collected at the beginning of the trial (time 0), while at 11 and 33 days, 5 fecal samples (1 pool/pen) were analyzed for each dietary treatment (20 samples/sampling time).

At each sampling time, the pooled fecal samples were transferred with a sterile spatula in an Eppendorf tube to be stored at −80 °C until analysis. Nucleic acid was extracted from fecal samples at each sampling point. Total DNA from the samples was extracted using the RNeasy Power Microbiome KIT (Qiagen, Milan, Italy) following the manufacturer’s instructions. One microliter of RNase (Illumina Inc., San Diego, CA, USA) was added to digest RNA in the DNA samples with an incubation of 1 h at 37 °C. DNA was quantified using the NanoDrop and standardized at 5 ng/μL. DNA directly extracted from fecal samples was used to assess the microbiota by the amplification of the V3–V4 region of the 16S rRNA and sequenced in paired-end mode (2X250) as elsewhere reported [41].

### 2.9. Bioinformatics and Statistical Analysis

The statistical analysis regarding bird performance, carcass traits, meat quality, and histomorphological findings was performed using the IBM SPSS Statistics package (IBM Corp. Released 2011. IBM SPSS Statistics for Windows, Version 20.0. Armonk, NY, USA: IBM Corp.). Normality of the data distribution and homogeneity of variances were assessed using the Shapiro–Wilk test and the Levene test, respectively. The experimental unit was the pen for growth performance, while the individual bird was considered for the slaughtering performance, meat quality traits, and histomorphological features. The collected animal performance and meat quality data were analyzed according to the general linear model (GLM) procedure, with the treatment as the main effect and the pen as the random effect. Multiple comparisons were performed using Tukey’s HSD test, when variances among groups were homogeneous, and Games-Howell test, when variances were not homogeneous.

Intestinal morphometric indices were analyzed by fitting a GLM. The model allowed the morphometric indices (Vh, Cd, and Vh/Cd, separately) to depend on 3 fixed factors (diet, intestinal segment, and interaction between diet and intestinal segment). Animal was included as a random effect to account for repeated measurements on the same bird. The interactions between the levels of the fixed factors were evaluated by pairwise comparisons. Histopathological scores were analyzed by Kruskal–Wallis test (post hoc test: Dunn’s multiple comparison test).

The 16S data were analyzed using QIIME 1.9.0 software [42], and the pipeline described [41]. Alpha diversity indices were calculated using the diversity function of the vegan package [43]. The diversity indices were further analyzed using the pairwise comparisons using Wilcoxon rank sum test to assess differences between the diets. A filtered operational taxonomic units (OTUs) table was generated at 0.1% abundance in at least 2 samples through QIIME. The table was then used to produce the principal component analysis (PCA) in R environment (www.r-project.org, accessed on 15 March 2021). The OTUs table displays the higher taxonomy resolution that was reached by the 16S data. OTUs table were used to perform Adonis and Anosim statistical tests in R environment. A generalized linear model was used in order to test the importance of continuous or discrete variables available for the birds (sampling time and diet) on the relative abundance of bacterial genera or family.

Significance was declared at *p* < 0.05. A statistical trend was considered for *p* < 0.10. Results were expressed as mean and pooled standard error of the mean (SEM).

## 3. Results

### 3.1. Growth Performance

Dietary BSF larvae fat and modified BSF fat inclusion did not affect the growth performance of the broiler chickens (Table 3), even if a statistical trend was observed for LW at day 33 (*p* = 0.096), ADFI (*p* = 0.062 and *p* = 0.074, respectively), and ADG (*p* = 0.080 and *p* = 0.095, respectively) during the grower-finisher period and the overall period, respectively, being more favorable for MBSF2 than other groups.

### 3.2. Blood Parameters

The blood traits of the birds were not affected by the dietary treatment (*p* > 0.05; Table 4).

### 3.3. Slaughter Performance and Meat Quality Traits

The slaughter performance, the pH_24,_ and the color of the breast and thigh muscles and their chemical composition were not affected by the dietary treatment, except for the total lipids of the breast meat with lower concentration in the MBSF1 group (*p* < 0.05; Table 5 and Table 6, respectively).

### 3.4. Gut Morphology

The effects of diet, gut segment, and interaction between diet and gut segment on the gut morphometric indices of the broiler chickens fed insect oil-based diets are reported in Table 7 and Table 8. In particular, only the intestinal segment significantly affected the Vh and the Vh/Cd (*p* < 0.001). On the contrary, there was no significant influence of diet and interaction between diet and gut segment (*p* > 0.05) on the gut morphometric indices (Table 8). Indeed, the duodenum showed greater Vh and Vh/Cd (*p* < 0.001) than the other gut segments, with morphometric indices being also greater (*p* < 0.001) in the jejunum when compared to the ileum (Table 8).

### 3.5. Histopathological Findings

**Figure 1:**
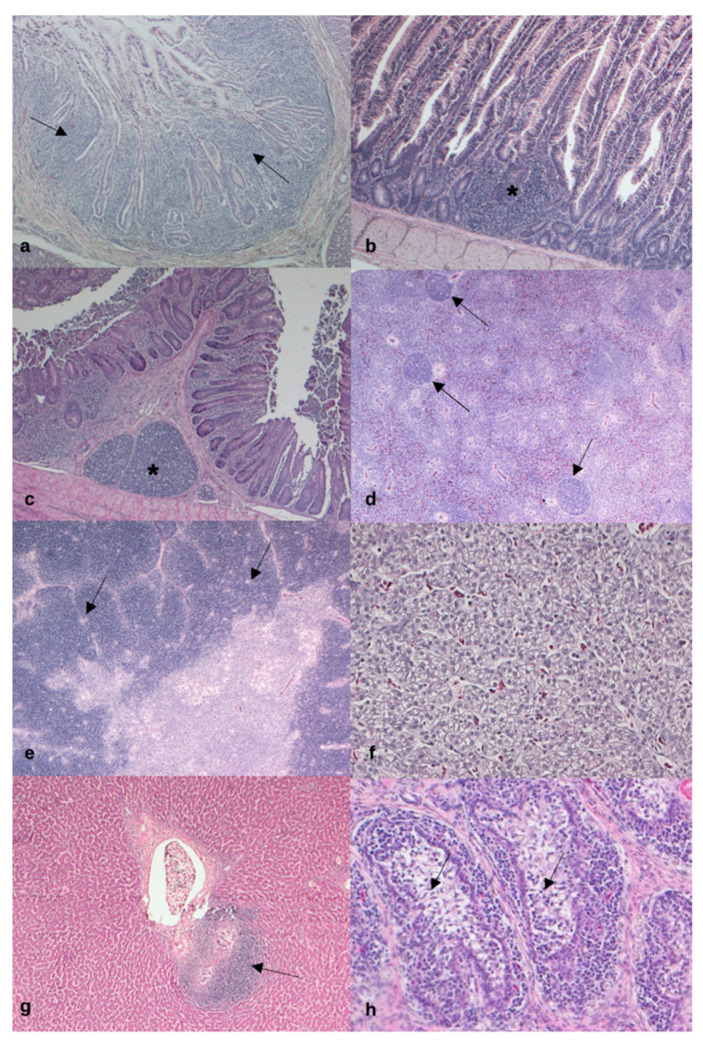
Main histopathological findings in the organs of the broiler chickens (n = 15/treatment). (**a**) Glandular stomach, mild lymphoplasmacytic infiltrates (black arrows), 5×, hematoxylin and eosin (HE). (**b**) Duodenum, mild lymphoplasmacytic infiltrates with lymphoid tissue hyperplasia (black asterisk), 5×, H-e. (**c**) Caecum, mild lymphoid tissue hyperplasia (black asterisk), 5×, H-e. (**d**) Mild white pulp hyperplasia (black arrows), 5×, H-e. (**e**) Thymus, mild cortical depletion (black arrows), 5×, H-e. (**f**) Liver, mild and multifocal vacuolar degeneration of the hepatocytes, 20×, H-e. (**g**) Liver, mild lymphoplasmacytic infiltrates (black arrow), 5×, H-e. (**h**) Bursa of Fabricius, mild follicular depletion (black arrows), 5×, H-e.

Mild histopathological alterations were observed in all groups. Mild, occasional lymphoplasmacytic infiltrates and lymphoid tissue hyperplasia were observed in the glandular stomach (proventriculus) (Figure 1a), duodenum, jejunum, and ileum (Figure 1b). Mild, multifocal lymphoid tissue hyperplasia was also detected in the caecum (Figure 1c). The spleen showed white pulp hyperplasia (Figure 1d), while cortical depletion was observed in the thymus (Figure 1e). Liver showed steatosis or vacuolar degeneration of the hepatocytes (Figure 1f), as well as lymphoplasmacytic infiltrates (Figure 1g). Follicular depletion was also detected in the bursa of Fabricius (Figure 1h). However, dietary BSF fat did not affect the severity of the observed histopathological alterations in any of the sampled organs (*p* > 0.05; Table 9).

### 3.6. Microbiota Characterization

**Figure 2:**
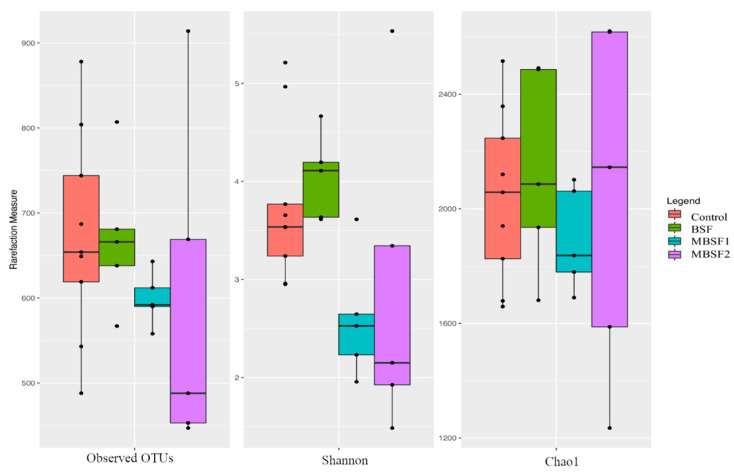
Boxplots to describe α-diversity measures of fecal microbiota of broiler chickens at the end of the experimental trial. BSF = diet with black soldier fly larvae fat; MBSF1 = diet with modified black soldier fly larvae fat type 1; MBSF2 = diet with modified black soldier fly larvae fat type 2.

After sequencing, the rarefaction analysis and the estimated sample coverage indicated that there was a satisfactory coverage of all the samples (ESC median value of 96.34%). By comparing the alpha-diversity values at the end of the experimental trial, it was possible to identify a significant decrease in complexity (Shannon index) of the microbiota only when comparing MBSF1 and MBSF2 vs. C and BSF fat inclusion (*p* < 0.05, Figure 2).

**Figure 3:**
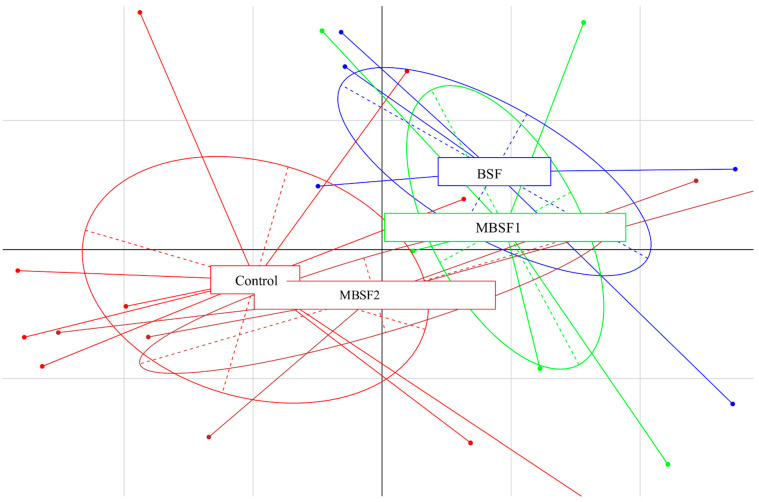
Principal component analysis (PCA) based on OTUs relative abundance of broiler chickens at the end of the experimental trial. BSF = diet with black soldier fly larvae fat; MBSF1 = diet with modified black soldier fly larvae fat type 1; MBSF2 = diet with modified black soldier fly larvae fat type 2.

On the contrary, the alpha-diversity values were unaffected by dietary treatments either at the beginning of the trial or at 11 days (*p* > 0.05). By taking into account the effect of the dietary BSF fat inclusion by principal component analysis (PCA) of the microbiota at genus or family level (Figure 3), it was possible to observe a shift in the microbiota composition at the end of the experimental trial only (ANOSIM statistic, R: 0.1913; *p* = 0.028). In particular, a clear separation of the C samples vs. BSF group was observed, while MBSF1 was similar to BSF and well separated from the control. On the contrary, MBSF2 did not provide separation when compared to the other dietary treatments (Figure 3).

**Figure 4:**
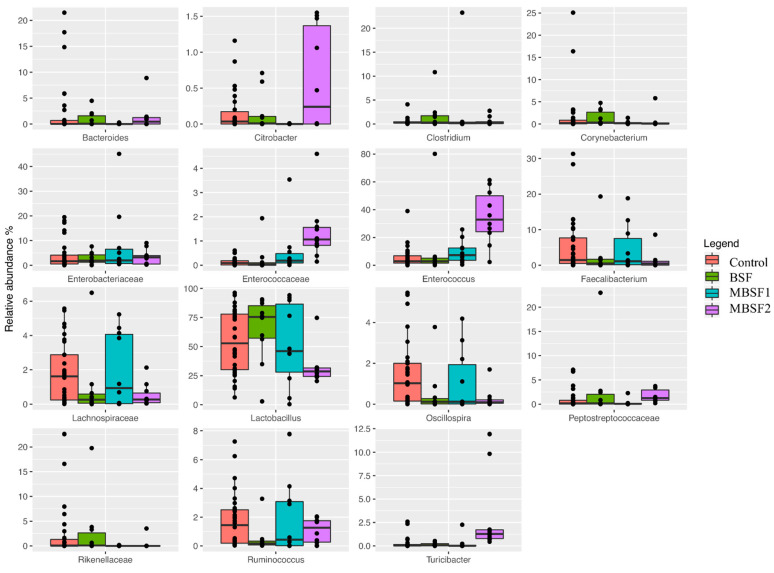
Boxplots showing the relative abundance at genus or family level of the OTUs differentially abundant based on generalized linear mix model (*p* ≤ 0.05) in fecal samples of broiler chickens independently of sampling time. BSF = diet with black soldier fly larvae fat; MBSF1 = diet with modified black soldier fly larvae fat type 1; MBSF2 = diet with modified black soldier fly larvae fat type 2.

A generalized linear model was used to test the importance of continuous or discrete variables available for the birds (sampling time and BSF larva fat inclusion) on the relative abundance of bacterial genera or families significantly different among the dietary treatments. Independently of the sampling time, it was possible to observe that BSF larva fat (either BSF, MBSF1, or MBSF2 treatments) increased the relative abundance of Bacteroides and Clostridium when compared to C group. In addition, this increase was more clearly observed when BSF larva fat was used (Figure 4). A microbial signature was also observed as a function of the insect oil inclusion for all the sampling times. In detail, BSF larva fat inclusion (BSF group) increased the relative abundance of *Clostridium*, *Lactobacillus*, and *Peptostreptococcaceae*, while reducing the presence of *Enterococcus*, *Faecalibacterium*, *Lachnospiraceae*, *Oscillospira*, and *Ruminococcus* (Figure 4). Furthermore, dietary MBSF1 inclusion increased the relative abundance of *Enterococcus* and reduced the presence of *Clostridium* and *Corynebacterium*. Finally, the use of MBSF2 increased the relative abundance of *Citrobacter*, *Enterococcaceae*, *Enterococcus*, *Peptostreptococcaceae*, and *Turicibacter* while decreasing the presence of *Corynebacterium*, *Faecalibacterium*, *Lachnospiraceae*, *Lactobacillus*, and *Rikenellaceae* (Figure 4).

## 4. Discussion

To our knowledge, no prior studies have examined the use of modified BSF larvae fat in broiler chicken feed, and currently, research on the suitability of BSF larvae fat as a feed additive in poultry diet is still scarce. Previously, the data suggested that the growth performance, nutrient digestibility, intestinal morphological features, and even quality of the carcass and meat are not adversely affected by insect fat additions or supplementations to the diet. However, the current study aimed to analyze the potential effects of BSF and MBSF fat used as feed additives on performance, gut health, and meat quality.

The results obtained in this study did not reveal significant differences on growing performance between the different groups in all periods of the present experiment. Until now, previous research studying the possibility of including insect fat in broiler chicken diets has led to controversial results as far as the performance parameters, carcass, and meat quality are concerned. The discrepancy between all the studies may be due to differences in age, breed, trial conditions and management, diet composition, and to the large variation in FA compositions of insect oils [44,45,46]. Schiavone et al. [4,5] and Sypniewski et al. [9], used BSF larvae fat to partially and totally replace SO and did not observe any adverse effects on growth performance in both broiler chickens and in young turkey poults. Comparing the effect of three fat sources including corn oil, coconut oil, and BSF larvae fat in broiler chickens, Kim et al. [47] did not show an effect on BW, ADG, or ADF at 15 and 30 days old but observed decreased FCR in the coconut oil and BSF larvae fat groups compared to that in the corn oil group. The authors attributed the improvement of FCR to the effect of medium chain FA rich fats, which improve nutrient digestion and absorption. As far as the utilization of *Tenebrio molitor* (TM) oil is concerned, a broiler trial that has been performed has shown that TM oil in substitution of SO decreased ADFI and FCR, without any effect on ADG [48]. The same authors reported that the use of 5% of TM or *Zophobas morio* fats in total substitution of SO in a 28-day trial did not affect the growth and feed efficiency of broiler chickens but showed differences in ADG between treatments for intermediate periods (14–21 days and 21–28 days). Similar conclusions on poultry growth were obtained by Benzertiha et al. [49] in a trial carried out on broiler chickens, where palm oil and poultry fat were totally substituted with TM oil. All the previous research confirmed the possibility of using insect fat in broiler chickens as an alternative to the conventional lipid sources.

The serum biochemical parameters give information about health status of the birds [50]. The results of this study suggested that the broiler chickens were within the physiological conditions and confirmed that BSF and MBSF larva fats did not affect the health status of the animals. Our results are in accordance with Schiavone et al. [4,5] and Sypniewski et al. [9], who did not report any significant difference in blood parameters of broiler chickens and turkey poults fed BSF larvae fat. Kim et al. [47] showed that BSF larvae fat decreased HDL cholesterol and total cholesterol in serum samples compared to coconut oil. However, BSF larvae fat did not affect serum levels of ALT, AST, triglyceride, or uric acid [47].

In the present study, dietary BSF larvae fat and MBSF larvae fat inclusion did not influence either the carcass traits or technological properties and chemical composition of meat. Nonetheless, all observed values as to pH, meat colors, and cooking loss of breast and thigh meats were within the acceptable range of meat characteristics [8]. This is in agreement with the previous results reported by Schiavone et al. [4,5] and Cullere et al. [8], who did not observe any effects on the studied traits in broiler chickens fed with BSF larvae meal in growing and finisher phases.

BSF larva and MBSF larva fat inclusion as feed additives did not affect the intestinal morphology, and the evolution of the morphological parameters along the different intestinal parts supports that the gut of the birds in this trial was in good condition for all treatments, including the control. Furthermore, the identification of a proximodistal decreasing gradient of the morphometric indices from the duodenum to the ileum is indicative of the preservation of the well-known, physiological gut development and absorption processes observed in poultry [41]. Similarly, the absence of significant histopathological alterations in the BSF fat-fed birds is also indicative that both BSF larva and MBSF larva fats do not impair the overall health status of the birds. All these findings are also in agreement with the previous research by Schiavone et al. [5] and Sypniewski et al. [9], where the BSF fat was tested as replacement of the soybean oil in finisher broiler chickens and turkey poultry nutrition.

The feed additive supplements in poultry diets is one of the strategies to manipulate gut microbiota and the immune system of the host in order to obtain better growth and health and to develop cost-effective feeding programs [2]. The glycerol monobutyrin in MBSF fat is a polar monoglyceride of butyric acid (C4: 0). Several studies have shown that SCFAs are able to control and reduce the development of pathogenic bacterial populations in the digestive tract of poultry [51]. The monoglycerides of butyric acid in MBSF fats can be preferred because they are easily absorbable and practically odorless. In particular, butyric acid has proven to be effective against colonization of the blind intestine by *Salmonella* spp. [18,52]. Butyric acid in the form of monoglyceride, compared to free acid, does not have unpleasant odors, and is more bioavailable [53]. According to some researchers, the addition of butyric acid derivatives to the broiler diet would reduce Salmonella enteritis infections [54] and would improve the growth performance of stressed animals [55]. For these reasons, these molecules are promising in order to reduce the use of antibiotics in poultry farming [56,57].

In relation to the microbial composition, *Citrobacter*, *Clostridium*, *Lactobacillus*, *Lachnospiraceae*, *Ruminococcus*, *Enterococcus*, *Peptostreptococcaceae*, and *Turicibacter* were the core microbiota. The majority of these taxa (*Clostridium*, L-*Ruminococcus*, and R-*Ruminococcus*) are characteristic members of the chicken microbiota [58,59,60], being also involved in the production of metabolites that are fundamental to the health status of the gut barrier. Regarding the modulation of the gut microbiota, we could observe an increase in the relative abundance of *Bacteroides* as a function of the oil inclusion, which is often associated with good performance [61]. In addition, a positive correlation was observed between *Bacteroides* and anti-inflammatory properties [62]. Moreover, BSF larvae fat inclusion also increased *Lactobacillus*, which has previously been associated with a potential for probiotics in chickens [63], and the protective role of *Lactobacillus* against pathogenic infection has been well-reported [64]. The ability of *Lactobacillus* to improve intestinal health could be due to its metabolic activity enabling reduction of the pH value. In this way, the gut environment becomes unsuitable for colonization by pathogenic microbes. Lactic-acid-producing bacteria isolated from chickens decrease pH value in caeca and improve the intestinal microenvironment, which becomes unsuitable for the activity and proliferation of pathogenic microbes [65]. Insect oil (especially BSF larvae and MBSF2) increased the presence of *Peptostreptococcaceae*; members of this taxa can produce organic acids that beneficially impact the healthy status of broilers [66,67]. A positive effect on the microbiota was observed by the reduction of *Clostridium* (especially by MBSF1 and MBSF2). It is well-known that members of the *Clostridium* genus can produce toxins that can cause inflammation, necrotic enteritis, or intestinal damage [68,69]. It is well-known that poultry represent an ecological niche for *Clostridium*, and our results suggest that modified insect oil can have a potentially antimicrobial effect on this taxa [70]. MBSF1 and MBSF2 inclusion also showed a positive effect on the reduction of Corynebacterium, which can cause disease in chickens [71,72]. Reducing microorganisms of this type is important as chickens’ excreta are the main source of contamination for other chickens, and in that light a reduction of this taxa is desirable.

## 5. Conclusions

The current study provides novel useful information on the use of BSF larvae fat and modified BSF larvae fat in broiler chicken diets. Dietary BSF larvae fat inclusion did not significantly influence growth performance, thus suggesting that these new alternative ingredients allow broilers to maintain required high growth standards. A shift in the microbiota composition was observed as a function of the diets. In particular, the use of BSF larvae fat with an increased content of monoglyceride, such as MBSF1 and MBSF2, reduced the presence of *Clostridium* and *Corynebacterium*, which can frequently cause infections in poultry. This can be suggestive of a healthy status of the broiler gut. The positive modulation of microbiota observed in BSF larvae fat-fed broilers is particularly relevant, because the inclusion of these novel ingredients could allow the reduction of antimicrobial use along with the associated phenomena of microbial resistance in poultry. Further research is needed to confirm the results of this study.

## Figures and Tables

**Table 1:** Ingredients and chemical composition of the starter and grower-finisher diets.

Ingredients (g/kg)		Starter Period (1–11 d)				Grower-Finisher Period (11–33 d)			
		C	BSF	MBSF1	MBSF2	C	BSF	MBSF1	MBSF2
Corn meal		508.8	508.8	508.8	508.8	536.1	536.1	536.1	536.1
Soybean meal 48		345.3	345.3	345.3	345.3	332.0	332.0	332.0	332.0
Corn gluten feed		54.0	54.0	54.0	54.0	35.0	35.0	35.0	35.0
Soybean oil		46.3	43.4	38.3	42.8	59.3	57.7	55.3	57.5
BSF fat		-	2.9	-	-	-	1.6	-	-
MBSF1		-	-	8.0	-	-	-	4.0	-
MBSF2		-	-	-	3.5	-	-	-	1.8
Calcium phosphate		5.3	5.3	5.3	5.3	3.5	3.5	3.5	3.5
Calcium carbonate		18.5	18.5	18.5	18.5	17.2	17.2	17.2	17.2
Sodium chloride		2.3	2.3	2.3	2.3	2.3	2.3	2.3	2.3
Sodium bicarbonate		1.3	1.3	1.3	1.3	1.3	1.3	1.3	1.3
L-lysine		5.1	5.1	5.1	5.1	3.8	3.8	3.8	3.8
DL-methionine		2.0	2.0	2.0	2.0	1.8	1.8	1.8	1.8
Threonine		1.9	1.9	1.9	1.9	1.5	1.5	1.5	1.5
3-phytase *		1.0	1.0	1.0	1.0	1.0	1.0	1.0	1.0
Vitamin and mineral premix ^a^		8.0	8.0	8.0	8.0	5.0	5.0	5.0	5.0
Choline chloride		0.2	0.2	0.2	0.2	0.2	0.2	0.2	0.2
Total		1000	1000	1000	1000	1000	1000	1000	1000
AME (kcal/kg) (calculated)		3000	3000	3000	3000	3100	3100	3100	3100
Chemical Composition (Analyzed)									
Dry matter (DM), %	89.6		89.7	90.0	89.7	90.1	90.0	89.8	90.2
Ash, % DM	6.32		6.19	6.39	6.62	6.12	6.25	6.45	6.47
Crude protein, % DM	23.0		23.0	23.0	23.0	21.5	21.5	21.5	21.5
Ether extract, %DM	6.41		6.86	6.28	6.07	7.96	8.18	7.72	7.28
Lysine, %	1.44		1.44	1.44	1.44	1.29	1.29	1.29	1.29
Methionine, %	0.56		0.56	0.56	0.56	0.51	0.51	0.51	0.51
Threonine, %	0.97		0.97	0.97	0.97	0.88	0.88	0.88	0.88
Calcium, %	0.96		0.96	0.96	0.96	0.87	0.87	0.87	0.87
Phosphorus, %	0.48		0.48	0.48	0.48	0.43	0.43	0.43	0.43

C = control diet; BSF = diet with black soldier fly larvae fat; MBSF1 = diet with modified black soldier fly larvae fat type 1; MBSF2 = diet with modified black soldier fly larvae fat type 2; AME= apparent metabolizable energy; * 3-phytase: E-300; natuphos bio/G500; ^a^ Mineral-vitamin premix: vitamin A (retinyl acetate), 12,500 IU; vitamin D3 (cholecalciferol), 3000 IU; vitamin E (DL-a-tocopheryl acetate), 60 IU; vitamin K (menadione sodium bisulfite), 1.02 mg; riboflavin, 2.0 mg; pantothenate, 8.0 mg; niacin, 6 mg; pyridoxine, 4 mg; folic acid, 0.5 mg; biotin, 0.10 mg; thiamine, 1.0 mg; vitamin B12, 20 mg; Mn, 120 mg; Zn, 80 mg; Fe, 52 mg; Cu, 15 mg; I, 1.5 mg; Se, 0.4 mg.

**Table 2:** Fatty acid profile of dietary fats and experimental diets (% of total FA).

Fatty Acid	Dietary Fats				Experimental Diets							
					Starter Diet				Grower-Finisher Diet			
	SO	BSF	MBSF1	MBSF2	C	BSF	MBSF1	MBSF2	C	BSF	MBSF1	MBSF2
C12:0	0.00	46.86	45.46	47.35	0.00	1.49	1.28	0.58	0.00	0.65	0.62	0.30
C14:0	0.08	9.87	9.80	10.06	0.10	0.40	0.33	0.22	0.00	0.22	0.19	0.13
C16:0	10.92	14.38	15.17	14.56	12.30	12.65	12.00	12.17	11.39	11.35	11.64	12.31
C16:1	0.09	2.78	2.92	2.77	0.16	0.17	0.00	0.00	0.00	0.00	0.15	0.16
C18:0	3.43	1.79	1.98	1.80	3.28	3.20	3.30	3.30	3.43	3.38	3.34	3.31
C18:1 n9 c	25.23	7.73	8.26	7.57	25.78	24.92	25.22	25.58	25.90	25.57	25.43	25.05
C18:2 n6	52.30	12.77	13.38	12.47	51.73	50.62	51.83	51.74	52.82	51.99	51.55	51.64
C18:3 n3	6.22	0.98	1.03	0.95	4.90	4.83	4.95	4.97	5.35	5.29	5.27	5.28
C20:0	0.31	0.07	0.00	0.00	0.35	0.33	0.36	0.36	0.38	0.37	0.34	0.33
C20:1	0.26	0.77	0.74	0.81	0.30	0.30	0-36	0.28	0.31	0.32	0.30	0.24
ΣSFA	14.95	74.24	73.40	74.99	16.20	18.32	17.27	16.83	15.19	16.16	16.31	16.63
ΣMUFA	25.75	11.58	11.98	11.30	26.40	25.53	25.57	25.85	26.21	25.88	26.09	25.61
ΣPUFA	58.74	14.00	14.60	13.62	57.25	56.01	57.14	57.30	58.58	57.95	57.58	57.62
Other FAs	1.19	1.74	2.02	0.91	0.76	0.80	0.37	0.59	0.42	0.67	0.76	0.92

SO = soybean oil; C = control diet; BSF = diet with black soldier fly larvae fat; MBSF1 = diet with modified black soldier fly larvae fat type 1; MBSF2 = diet with modified black soldier fly larvae fat type 2; SFA = saturated fatty acid; MUFA = monounsaturated fatty acid; PUFA = polyunsaturated fatty acid.

**Table 3:** Effect of black soldier fly larvae fat on growth performance of broiler chickens (n = 5 pens/treatment; 10 birds/pen).

Items	Experimental Diets				SEM	*p*-Value
	C	BSF	MBSF1	MBSF2		
LW at 1 d, g	45.2	45.3	45.3	45.3	0.074	0.983
LW at 11 d, g	318.3	320.7	313.8	317.3	2.917	0.887
LW at 33 d, g	2002.5	1937.2	1920.5	2067.8	23.636	0.096
ADG 1–11 d, g	24.8	25.0	24.4	24.7	0.263	0.882
ADG 11–33 d, g	76.6	73.5	73.0	79.6	1.042	0.080
ADG 1–33 d, g	59.3	57.3	56.8	61.3	0.716	0.096
ADFI 1–11 d, g	27.4	28.6	27.0	28.0	0.343	0.390
ADFI 11–33 d, g	101.3	97.5	98.3	113.1	2.393	0.062
ADFI 1–33 d, g	77.5	74.7	75.2	86.0	1.775	0.074
FCR 1–11 d	1.11	1.13	1.10	1.06	0.010	0.172
FCR 11–33 d	1.26	1.31	1.29	1.34	0.015	0.325
FCR 1–33 d	1.25	1.28	1.27	1.32	0.011	0.245

SO = soybean oil; C = control diet; BSF = diet with black soldier fly larvae fat; MBSF1 = diet with modified black soldier fly larvae fat type 1; MBSF2 = diet with modified black soldier fly larvae fat type 2 LW: live weight (g); ADG: average daily gain (g/d); ADFI: average daily feed intake (g/d); FCR: feed conversion ratio; SEM: pooled standard error of the mean.

**Table 4:** Effect of black soldier fly larvae fat on blood traits of broiler chickens (n = 15/treatment).

Items	Experimental Diets				SEM	*p*-Value
	C	BSF	MBSF1	MBSF2		
ALB (g/dL)	1.35	1.38	1.39	1.45	0.020	0.441
ALT (UI/L)	2.80	2.57	2.67	2.90	0.063	0.264
AST (UI/L)	319.5	330.8	317.8	339.8	6.042	0.546
ALP (UI/L)	5492.6	5705.6	6519.0	5983.0	379.55	0.835
GGT (UI/L)	29.4	29.6	29.9	29.7	1.294	0.999
Total cholesterol (mg/dL)	113.5	116.6	115.1	119.7	2.096	0.765
HDL-cholesterol (mg/dL)	85.1	90.9	89.1	92.6	1.651	0.424
LDL-cholesterol (mg/dL)	29.1	26.7	26.0	27.8	1.112	0.786
Triglycerides (mg/dL)	41.8	40.6	40.7	37.0	1.435	0.669
Uric acid (mg/dL)	4.98	5.00	4.74	4.89	0.174	0.955
Creatinine (mg/dL)	0.09	0.10	0.08	0.10	0.005	0.692
Total protein (g/dL)	3.22	3.13	3.23	3.34	0.048	0.469
Phosphorus (mg/dL)	90.6	70.1	63.9	94.4	5.404	0.125
Chlorine (mmol/L)	118.6	117.9	116.6	125.2	1.529	0.171
Potassium (mmol/L)	6.81	7.04	6.64	7.66	0.156	0.088
Magnesium (mEq/L)	6.01	6.03	5.80	5.95	0.107	0.885
Iron (µg/dL)	99.7	88.3	96.4	90.8	2.618	0.401
Sodium (mmol/L)	154.2	165.1	163.6	174.9	3.428	0.194
Calcium (mg/dL)	48.7	48.7	53.2	49.6	0.749	0.121

C = control diet; BSF = diet with black soldier fly larvae fat; MBSF1 = diet with modified black soldier fly larvae fat type 1; MBSF2 = diet with modified black soldier fly larvae fat type 2; SEM: pooled standard error of the mean; ALB = Albumin; AST = aspartate aminotransferase; ALT = alanine aminotransferase; ALP = alkaline phosphatase; GGT = gamma-glutamyl transferase; HDL = high-density lipoprotein cholesterol; LDL = low-density lipoprotein cholesterol.

**Table 5:** Effect of black soldier fly larvae fat on slaughter performance of broiler chickens (n = 15/treatment).

Items	Experimental Diets				SEM	*p*-Value
	C	BSF	MBSF1	MBSF2		
Slaughter weight (SW), g	2032.2	2021.8	2017.6	2108.8	19.806	0.327
Carcass weight (CCW), g	1396.9	1382.5	1364.8	1442.4	15.336	0.328
Slaughter yield, % SW	68.8	68.3	67.6	68.4	0.249	0.439
Breast, % CCW	31.8	32.2	32.5	33.0	0.256	0.389
Thigh, % CCW	31.3	30.5	30.6	30.8	0.305	0.755
Spleen, % SW	0.11	0.11	0.11	0.11	0.004	0.985
Liver, % SW	2.04	2.02	2.02	2.06	0.020	0.886
Bursa of Fabricius, % SW	0.24	0.28	0.27	0.30	0.009	0.222
Heart, % SW	0.66	0.70	0.68	0.68	0.017	0.891
Proventriculus (glandular stomach), % SW	0.37	0.36	0.36	0.37	0.008	0.998
Gizzard (muscular stomach), % SW	1.38	1.38	1.54	1.32	0.047	0.400
Intestine, % SW	4.87	5.37	5.13	5.14	0.153	0.727

C = control diet; BSF = diet with black soldier fly larvae fat; MBSF1 = diet with modified black soldier fly larvae fat type 1; MBSF2 = diet with modified black soldier fly larvae fat type 2; SEM: pooled standard error of the mean.

**Table 6:** Effect of black soldier fly larvae fat on meat quality traits and chemical composition of broiler chickens (n = 15/treatment).

Items	Experimental Diets				SEM	*p*-Value
	C	BSF	MBSF1	MBSF2		
Breast meat						
pH	5.99	5.99	5.97	6.04	0.013	0.288
L	52.9	52.6	52.2	53.2	0.366	0.814
a*	0.72	0.71	0.61	0.90	0.179	0.951
b*	12.5	13.0	11.9	12.3	0.275	0.552
	Chemical Composition (%)					
Water	24.3	24.6	24.6	24.1	0.095	0.231
Ash	1.24	1.21	1.25	1.19	0.017	0.576
Crude protein	22.7	22.5	22.4	22.5	0.138	0.940
Total lipids	1.45 ^a^	1.33 ^ab^	1.16 ^b^	1.44 ^a^	0.040	0.029
Thigh meat						
pH	6.18	6.16	6.17	6.16	0.012	0.966
	Chemical Composition (%)					
Water	28.2	27.3	27.7	28.6	0.337	0.586
Ash	1.04	1.14	1.04	1.08	0.018	0.132
Crude protein	18.9	19.2	18.4	18.9	0.115	0.114
Total lipids	9.28	8.22	8.98	7.90	0.308	0.357

C = control diet; BSF = diet with black soldier fly larvae fat; MBSF1 = diet with modified black soldier fly larvae fat type 1; MBSF2 = diet with modified black soldier fly larvae fat type 2; SEM: pooled standard error of the mean. ^a,b^ Different superscripts within a row indicate significant differences (*p* < 0.05).

**Table 7:** Effects of diet, intestinal segment, and interaction between diet and intestinal segment on the intestinal morphometric indices of the broiler chickens (n = 15/treatment).

Index	Fixed Effect	d.f. ^3^	F	*p*-Value ^4^
Vh (mm)	Diet ^1^	3	0.166	0.919
	Intestinal segment ^2^	2	155.045	<0.001
	Diet × Intestinal segment	6	0.820	0.556
Cd (mm)	Diet	3	0.888	0.448
	Intestinal segment	2	0.133	0.875
	Diet × Intestinal segment	6	1.220	0.298
Vh/Cd (mm/mm)	Diet	3	0.872	0.457
	Intestinal segment	2	143.538	<0.001
	Diet × Intestinal segment	6	0.547	0.772

Vh: villus height; Cd: crypt depth; Vh/Cd: villus height to crypt depth ratio; ^1^ 4 dietary treatments: C = control diet; BSF = diet with black soldier fly larvae fat; MBSF1 = diet with modified black soldier fly larvae fat type 1; MBSF2 = diet with modified black soldier fly larvae fat type 2; ^2^ 3 intestinal segments: duodenum, jejunum, and ileum; ^3^ Degrees of freedom; ^4^ Statistical significance: *p* < 0.05.

**Table 8:** Least square means of intestinal morphometric indices in broilers in relation to diet and intestinal segment (n = 15/treatment).

Index	Fixed Effect	Effect Levels	Least Square Mean ^1^	SEM
Vh (mm)	Diet ^2^	C	2.28	0.08
		BSF	2.28	
		MBSF1	2.23	
		MBSF2	2.30	
	Intestinal segment ^3^	DU	3.24 ^a^	0.07
		JE	1.95 ^b^	
		IL	1.63 ^c^	
Cd (mm)	Diet ^2^	C	0.17	0.01
		BSF	0.18	
		MBSF1	0.18	
		MBSF2	0.18	
	Intestinal segment ^3^	DU	0.18	0.00
		JE	0.18	
		IL	0.18	
Vh/Cd (mm/mm)	Diet ^2^	C	13.13	0.46
		BSF	12.33	
		MBSF12	12.31	
		MBSF	12.99	
	Intestinal segment ^3^	DU	18.12 ^a^	0.40
		JE	10.8 ^b^	
		IL	9.15 ^c^	

SEM= pooled standard error of the mean; Vh= villus height; Cd= crypt depth; Vh/Cd= villus height to crypt depth ratio; ^1^ Means with different superscript letters (a, b, c) within the same column per fixed effect (i.e., diet, intestinal segment) differ significantly (*p* < 0.05); ^2^ C = control diet; BSF =diet with black soldier fly larvae fat; MBSF1 = diet with modified black soldier fly larvae fat type 1; MBSF2 = diet with modified black soldier fly larvae fat type 2; ^3^ DU = duodenum; JE = jejunum; IL = ileum.

**Table 9:** Effects of dietary BSF larvae fat inclusion on the histopathological scores of the broiler chickens (n = 15/treatment).

Items	Experimental Diets				SEM	*p*-Value
	C	BSF	MBSF1	MBSF2		
Spleen	0.53	0.27	0.27	0.07	0.06	0.072
Liver	0.23	0.27	0.33	0.27	0.06	0.883
Thymus	0.06	0	0.06	0	0.03	0.591
Bursa of Fabricius	0.60	0.77	0.67	0.5	0.08	0.650
Glandular stomach	1.87	1.80	1.93	1.87	0.08	0.873
Gut	2.40	2.20	2.00	1.73	0.16	0.531

C = control diet; BSF = diet with black soldier fly larvae fat; MBSF1 = diet with modified black soldier fly larvae fat type 1; MBSF2 = diet with modified black soldier fly larvae fat type 2; SEM: pooled standard error of the mean.

## Data Availability

The data that support the findings of this study are available upon request.
